# Developing a mortality risk prediction model using data of 3663 hospitalized COVID-19 patients: a retrospective cohort study in an Egyptian University Hospital

**DOI:** 10.1186/s12890-023-02345-3

**Published:** 2023-02-07

**Authors:** Sahar Kandil, Ayman I. Tharwat, Sherief M. Mohsen, Mai Eldeeb, Waleed Abdallah, Amr Hilal, Hala Sweed, Mohamed Mortada, Elham Arif, Tarek Ahmed, Ahmed Elshafie, Tarek Youssef, Mohamed Zaki, Yasmin El-Gendy, Essam Ebied, Safwat Hamad, Ihab Habil, Hany Dabbous, Amr El-Said, Yasser Mostafa, Samia Girgis, Ossama Mansour, Ali El-Anwar, Ashraf Omar, Ayman Saleh, Mahmoud El-Meteini

**Affiliations:** 1 Department of Community, Environmental, and Occupational Medicine, Faculty of Medicine, Ain Shams University, 38 Ramses St., Abbassia Square, Cairo, 1156 Egypt; 2 Department of Anaesthesia, Intensive Care and Pain Management, Faculty of Medicine, Ain Shams University, Cairo, Egypt; 3 Department of General Surgery, Faculty of Medicine, Ain Shams University, Cairo, Egypt; 4 Department of Internal Medicine, Faculty of Medicine, Ain Shams University, Cairo, Egypt; 5 Department of Geriatric Medicine and Gerontology, Faculty of Medicine, Ain Shams University, Cairo, Egypt; 6 Department of Paediatrics, Faculty of Medicine, Ain Shams University, Cairo, Egypt; 7 Department of Scientific Computing, Faculty of Computer and Information Sciences, Ain Shams University, Cairo, Egypt; 8 Department of Hepatology and Infectious Diseases, Faculty of Medicine, Ain Shams University, Cairo, Egypt; 9 Department of Chest Diseases, Faculty of Medicine, Ain Shams University, Cairo, Egypt; 10 Department of Clinical Pathology, Faculty of Medicine, Ain Shams University, Cairo, Egypt; 11 Department of Otolaryngology and Head and Neck Surgery, Faculty of Medicine, Ain Shams University, Cairo, Egypt; 12 Department of Cardiology, Faculty of Medicine, Ain Shams University, Cairo, Egypt; 13 Department of Hepatobiliary Surgery and Liver Transplantation, Faculty of Medicine, Ain Shams Centre for Organ Transplantation (ASCOT),, Ain Shams University, Cairo, Egypt

**Keywords:** COVID-19, In-hospital mortality, Mortality predictors, Prognostic model, Comorbidities, Survival, Obesity, Smoking

## Abstract

**Purpose:**

Since the declaration of COVID-19 as a pandemic, a wide between-country variation was observed regarding in-hospital mortality and its predictors. Given the scarcity of local research and the need to prioritize the provision of care, this study was conducted aiming to measure the incidence of in-hospital COVID-19 mortality and to develop a simple and clinically applicable model for its prediction.

**Methods:**

COVID-19-confirmed patients admitted to the designated isolation areas of Ain-Shams University Hospitals (April 2020–February 2021) were included in this retrospective cohort study (*n* = 3663). Data were retrieved from patients’ records. Kaplan–Meier survival and Cox proportional hazard regression were used. Binary logistic regression was used for creating mortality prediction models.

**Results:**

Patients were 53.6% males, 4.6% current smokers, and their median age was 58 (IQR 41–68) years. Admission to intensive care units was 41.1% and mortality was 26.5% (972/3663, 95% CI 25.1–28.0%). Independent mortality predictors—with rapid mortality onset—were age ≥ 75 years, patients’ admission in critical condition, and being symptomatic. Current smoking and presence of comorbidities particularly, obesity, malignancy, and chronic haematological disorders predicted mortality too. Some biomarkers were also recognized. Two prediction models exhibited the best performance: a basic model including age, presence/absence of comorbidities, and the severity level of the condition on admission (Area Under Receiver Operating Characteristic Curve (AUC) = 0.832, 95% CI 0.816–0.847) and another model with added International Normalized Ratio (INR) value (AUC = 0.842, 95% CI 0.812–0.873).

**Conclusion:**

Patients with the identified mortality risk factors are to be prioritized for preventive and rapid treatment measures. With the provided prediction models, clinicians can calculate mortality probability for their patients. Presenting multiple and very generic models can enable clinicians to choose the one containing the parameters available in their specific clinical setting, and also to test the applicability of such models in a non-COVID-19 respiratory infection.

**Supplementary Information:**

The online version contains supplementary material available at 10.1186/s12890-023-02345-3.

## Background

Corona virus disease 2019 (COVID-19) caused by the novel SARS-CoV-2 virus was first reported in Wuhan city in China, December 2019. By March 11, 2020, COVID-19 was declared a pandemic after spreading to 114 countries [1]. Globally until 20 August 2022, there were 591,683,619 cases fulfilling the WHO case definition criteria of confirmed COVID-19 infection and including 6,443,306 deaths [2]. Egypt reported its first case on February 14th, 2020, and the numbers have been rising ever since. On August 20th, 2022, the number of documented patients was 515,198 with a death toll of 24,786 [3]. COVID-19 demonstrated a clinically diverse manifestation ranging from asymptomatic presentation to critical illness with severe pneumonia, acute respiratory distress syndrome, respiratory failure, or multiple organ failure. The common symptoms are fever, cough, dyspnoea, and altered/diminished taste/smell sensation; and most cases showed a favourable clinical course [4]. Evidence of extrapulmonary involvement was also demonstrated [5]. Reports showed an increased risk of death for older patients with pre-existing comorbidities, presence of ground-glass opacity in chest X-ray, and the potential of some blood biomarkers as early predictors of disease severity and mortality [4, 6–10].

Many challenges are still present that mandate further research in this novel disease. First, most of the evidence regarding the available therapeutic options for COVID-19 has very low or moderate certainty level [11]. Second, new strains are emerging worldwide with more variants having potential evolutional advantage over their ancestral types and could present a large global threat [12]. Third, in-hospital mortality and the factors predicting it varied widely (pooled estimate 15–55%) within different countries and healthcare settings [13, 14]. Fourth, despite the development of several prognostic models that predict in-hospital COVID-19 mortality, many were either based mainly on laboratory data [15, 16], or built using smaller sample of only severe patients [17]. Still there is a need for a model that is simple and practically useful in clinical settings.

In Egypt, the calculated case fatality rate according to the available data was 5.65%. The reported in-hospital mortality ranged between 18.9% (28/148) [18] and 24.4% (39/160) [19] based on studies conducted on a limited number of patients. Ain Shams University Hospitals (ASUHs), as one of the largest university hospitals in Egypt (~ 3500 beds), started to dedicate isolation areas for COVID-19 patients at the beginning of April 2020. With the surge of epidemic in June, the isolation capacity was expanded to also encompass patients referred from other healthcare facilities. Given the wide variability of reported in-hospital mortality and its predictors, the persistence of the epidemic, and the scarcity of local research, this study was conducted in ASUHs aiming to measure the incidence of in-hospital COVID-19 mortality and to identify its predictors, then to develop a mortality prediction model. This knowledge could help prioritize the provision of care to improve patients’ outcome.

## Methods

### Study design, setting, and group

This retrospective cohort study was conducted in the designated isolation areas of ASUHs including buildings of El-Obour, Geriatrics, and Field hospitals in addition to dedicated wards in Paediatrics, Surgery, and Medicine hospitals with a maximum capacity of ~ 450 beds. All patients admitted to isolation areas from April 2020 to the end of February 2021 were included in this study. They all have a laboratory confirmed diagnosis of COVID-19 based on real-time reverse-transcriptase–polymerase-chain-reaction (RT–PCR) test.

### Data collection

Although paper-based patients’ records were still used in our hospitals, a special electronic database was designed to collect data in the isolation areas to reduce paperwork and enhance infection prevention and control practice. Data retrieved for this study were the demographics and clinical data including age, gender, smoking status, medical history, symptoms, signs, comorbidities, baseline laboratory biomarkers, chest high resolution computed tomography (HRCT) reports, and dates of admission and discharge with the discharge status. The outcome variable was the in-hospital mortality as recorded in patients’ medical records on discharge.

### Definitions

Patients’ condition on admission regarding the disease severity was classified according to Ain Shams University Hospitals Consensus Statement on Management of Adult COVID-19 Patients [20]. Patients were categorized as asymptomatic [COVID-19 RT-PCR positive without clinical manifestations attributed to COVID-19], mild [symptomatic without chest HRCT evidence of COVID-19 pneumonia], moderate [symptoms of non-severe pneumonia (e.g., fever, cough, dyspnoea) and HRCT findings of COVID 19 pneumonia and/or abnormal biomarkers (D-dimer < 1mcg/mL, absolute lymphopenia < 800/µL, ferritin < 500 ng/mL, normal liver function)], severe [signs of severe pneumonia (e.g. respiratory rate > 30 breaths/minute, severe respiratory distress, or SpO2 < 93% on room air) and HRCT findings of COVID 19 pneumonia], and critical [respiratory failure necessitating mechanical ventilation, shock, sepsis, or other organ failure that requires management in intensive care unit (ICU)].

Radiological evidence of COVID-19 pneumonia in the HRCT was determined according to the COVID-19 Reporting and Data System (CO-RADS) staging of the level of suspicion into no, low, intermediate, high, and very high [21].

Comorbidities included hypertension, diabetes, ischemic and other heart diseases, obesity (body mass index > 30), chronic obstructive pulmonary disease (COPD) and other lung diseases, chronic kidney disease (CKD), cerebrovascular disease (stroke/CNS disease), chronic liver disease, malignancy, haematological disorders, immunological disorders, surgery, transplantation, and pregnancy.

Regarding haematological biomarkers: reference range for total leucocytic count is 4000–11 000/L and for platelet count is 150,000–450,000/L. For haemoglobin levels, anaemia and severe anaemia were considered with levels 8– < 13 g/dL and < 8 g/dL respectively in adult males, 7– < 11 g/dL and < 7 g/dL respectively in adult females, and 8– < 12 g/dL and < 8 g/dL respectively in patients < 15 years of age [22].

### Statistical analysis

For description, median and interquartile range (IQR) were calculated for quantitative variables and frequencies and percentages for categorical variables. Incidence proportion and hazard of mortality were calculated as the number of non-survivors divided by either the total number of patients or by the total patient-days respectively. For building prognostic models, mortality predictor variables were first determined.

#### Determination of mortality predictors

Bivariate Cox proportional hazard regression was used to test the effect of each predictor variable on mortality risk. To determine the independent mortality predictors, multivariable Cox proportional hazard regression models were constructed based on variables that were significant in bivariate analysis. Presence of comorbidities was first tested in multivariable Cox regression as simple dichotomous yes/no exposure variable to enable its further use in building of the prognostic models. Then, the effect of specific comorbidity and specific symptom was estimated after correction of each comorbidity or symptom separately by age, gender, and smoking status (Cox regression Model-1). Further correction by the severity of the condition on admission, and the presence of other comorbidities or symptoms was done (Cox regression Model-2) to disentangle the specific and independent mortality predictors. Variables tested were those with *P*-value < 0.05 on bivariate analysis. Adjusted hazard ratio (HR) and 95% confidence interval (CI) were calculated for each predictor variable. This two-step detailed analysis for an exhaustive list of comorbidities and symptoms was particularly made for two reasons first, to verify the existing evidence shown in literature after accounting for the basic confounders that usually considered in most research (Model-1). Second, to add to the evidence regarding the controversial issue of their independence as mortality risk factors (Model-2). Blood biomarkers were also tested for their association with the risk of mortality after accounting for age, gender, and smoking status using Cox proportional hazard regression. Kaplan–Meier method was used for calculating the cumulative survival between comparison groups of the predictor variables. Effect estimates, 95% CI, and exact *P*-values were presented.

#### Building of prognostic mortality prediction models

Prognostic models were built by the calculation of the predicted probability of death using multivariable logistic regression. Models were intended to be simple and containing the least possible number of relevant variables. The tested models were first, a basic model containing age (in years), severity of patients’ condition on admission, and the presence of comorbidities. These variables were chosen based on being significantly associated with higher risk of mortality. Then, the other models were built by adding each biomarker (numerical variable) to the basic one. The tested biomarkers were those showed significant association with the risk of mortality in the stage of determination of predictor variables. Additionally, models for testing the specific contribution of each comorbidity were built through replacing the “presence of comorbidity” variable in the basic model with each comorbidity variable that showed significance with mortality risk in Model-2. Addition of the smoking status to the basic model was also tested. Assessment of models’ performances was conducted by the receiver operating characteristic (ROC) analysis for their calculated predicted probability of death. Models with the best performance were presented regarding the area under the ROC curve (AUC and 95% CI). Sensitivity, specificity, positive and negative predictive values, overall accuracy (% correct), and balanced accuracy corresponding to the 50% prediction probability were also presented. Model calibration was performed visually by plotting the observed proportions of mortality events against the predicted risks for 10 equal-sized risk groups, and also by Hosmer–Lemeshow (HL) goodness of fit test. A small *P*-value for HL test indicates poor model fit for the data. All analyses were performed using SPSS version 25.

### Ethical statement

This study was approved by Ain Shams University Faculty of Medicine Research Ethics Committee (approval number FWA 00,017,585). This study was performed in accordance with the ethical standards of the Declaration of Helsinki, 1964 and its later amendments. All patients’ data were taken anonymously from their medical records and no identifying information was presented. The need for informed consent was waived by Ain Shams University Faculty of Medicine Research Ethics Committee.

## Results

### The study group and mortality rate

**Fig. 1:**
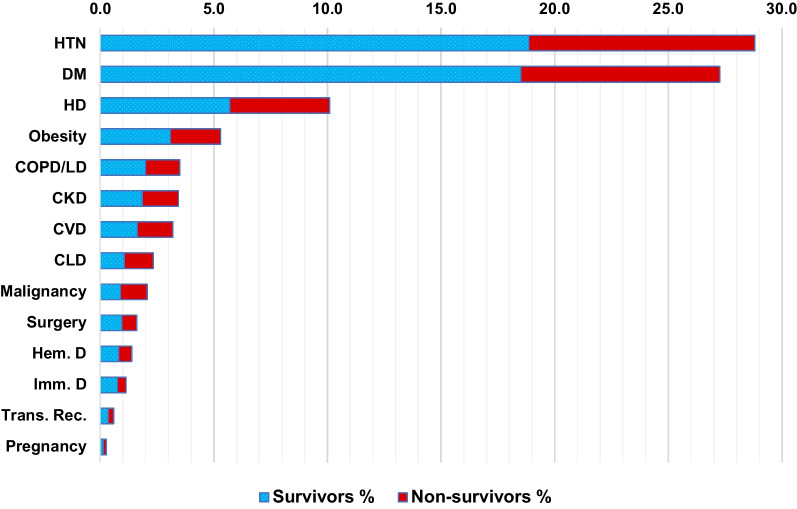
Preexisting comorbidities: percentages of survivors and non-survivors. Abbreviations: *HTN* Hypertension, *DM* Diabetes mellitus, *HD* Heart disease, *COPD/LD* chronic obstructive pulmonary disease/other lung diseases, *CKD* Chronic kidney disease, *CVD* Cerebrovascular disease, *CLD* Chronic liver disease, *Hem. D* Hematological disorders, *Imm. D* Immunological disorders, *Trans. Rec*. Transplant recipients

**Fig. 2:**
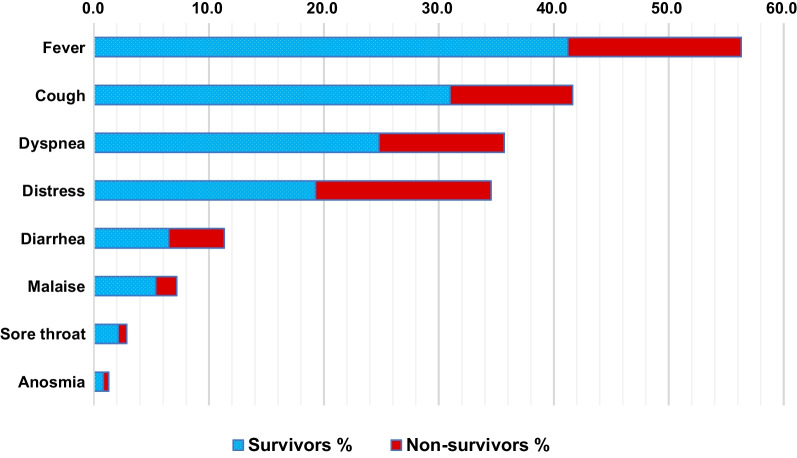
Common clinical presentation: percentages of survivors and non-survivors

This study included 3663 COVID-19 confirmed patients admitted to ASUHs isolation areas, of them 41.1% (1507/3663) were admitted to the ICU. Median age was 58 years (IQR 41–68 years), males were 53.6% (1965/3663), and the current and former smokers were 4.6% (170/3663) and 2.0% (74/3663) respectively. Median hospital stay was 8 days (IQR 4–12 days) and patients who stayed for one day or less were 6.3% (230/3663). Conditions on admission were severe and critical among two thirds of patients [39.5% (1447/3663) and 25.6% (937/3663) respectively] and patients that first presented with complications were 5.0% (182/3663). Comorbidities were observed in 45.0% (1649/3663) of patients (Table 1) and, in order of frequency, they were hypertension (28.8%, 1055/3663), diabetes (27.3%, 999/3663), heart disease [other than ischemic (5.8%, 213/3663) and ischemic (4.3%, 156/3663)], obesity (5.3%, 194/3663), and CKD (3.4%, 126/3663) (Fig. 1 and Additional file 1: Table S1). Patients commonly presented with fever (56.3%, 2062/3663), cough (41.6%, 1525/3663), dyspnoea (35.7%, 1307/3663), respiratory distress (34.5%, 1265/3663), diarrhoea (11.3%, 415/3663), and malaise (7.2%, 263/3663). Less common symptoms included sore throat (2.9%, 105/3663), anosmia (1.3%, 47/3663), vomiting (0.3%, 11/3663), ageusia (0.2%, 9/3663), and abdominal pain (0.2%, 8/3663). (Fig. 2 and Additional file 1: Table S2).

**Table 1:** Mortality by demographics and clinical characteristics: bivariate analysis (n = 3663)

	Total	Mortality	Unadjusted HR^a^ (95% CI)	*P*-value
	*n* = 3663	*n* = 972 (26.5) 95% CI (25.1–28.0%)		
	*n* (column%)	*n* (column%)		
Age: median (IQR)	58 (41–68)	66 (55–75)		** < 0.001**
< 15	165 (4.5)	32 (3.3)	Ref	
15–34	498 (13.6)	61 (6.3)	0.72 (0.47–1.10)	0.125
35–54	912 (24.9)	141 (14.5)	0.82 (0.56–1.21)	0.322
55–74	1618 (44.2)	490 (50.4)	1.55 (1.08–2.22)	**0.016**
≥ 75	470 (12.8)	248 (25.5)	2.91 (2.03–4.21)	** < 0.001**
Gender				
Female	1698 (46.4)	438 (45.1)	Ref	
Male	1965 (53.6)	534 (54.9)	1.05 (0.93–1.19)	0.440
Tobacco use				
Never	3419 (93.3)	877 (90.2)	Ref	
Former	74 (2.0)	28 (2.9)	1.29 (0.88–1.88)	0.194
Current	170 (4.6)	67 (6.9)	1.70 (1.32–2.18)	** < 0.001**
Condition on admission				
Asymptomatic/Mild	368 (10.0)	29 (3.0)	Ref	
Moderate	911 (24.9)	97 (10.0)	1.64 (1.08–2.48)	**0.020**
Severe	1447 (39.5)	238 (24.5)	2.48 (1.69–3.65)	** < 0.001**
Critical	937 (25.6)	608 (62.6)	10.14 (6.99–14.72)	** < 0.001**
Comorbidities				
No	2014 (55.0)	384 (39.5)	Ref	
Yes	1649 (45.0)	588 (60.5)	1.79 (1.57–2.03)	** < 0.001**
Patients presented in complications				
No	3481 (95.0)	858 (88.3)	Ref	
Yes	182 (5.0)	114 (11.7)	2.73 (2.24–3.31)	** < 0.001**
Hospital stay: median (IQR)	8 (4–12)	7 (4–12)		**0.010**
> 1Day	3433 (93.7)	863 (88.8)	Ref	
≤ 1Day	230 (6.3)	109 (11.2)	2.68 (2.05–3.51)	** < 0.001**

*HR* Hazard ratio, *CI* Confidence interval

Bold indicates statistical significance

^a^Bivariate Cox regression analysis was used

Mortality was 26.5% (972/3663, 95% CI 25.1%–28.0%) and 64.5% (972/1507, 95% CI 62.1–66.9%) among the total and ICU admitted patients respectively; and the daily hazard was 3.0% (972 mortality events/32834 patient-days, 95% CI 2.8–3.2%) and 6.9% (972 mortality events/14027 patient-days, 95% CI 6.5–7.4%) respectively.

### Mortality predictors

#### Demographics and clinical characteristics

**Fig. 3:**
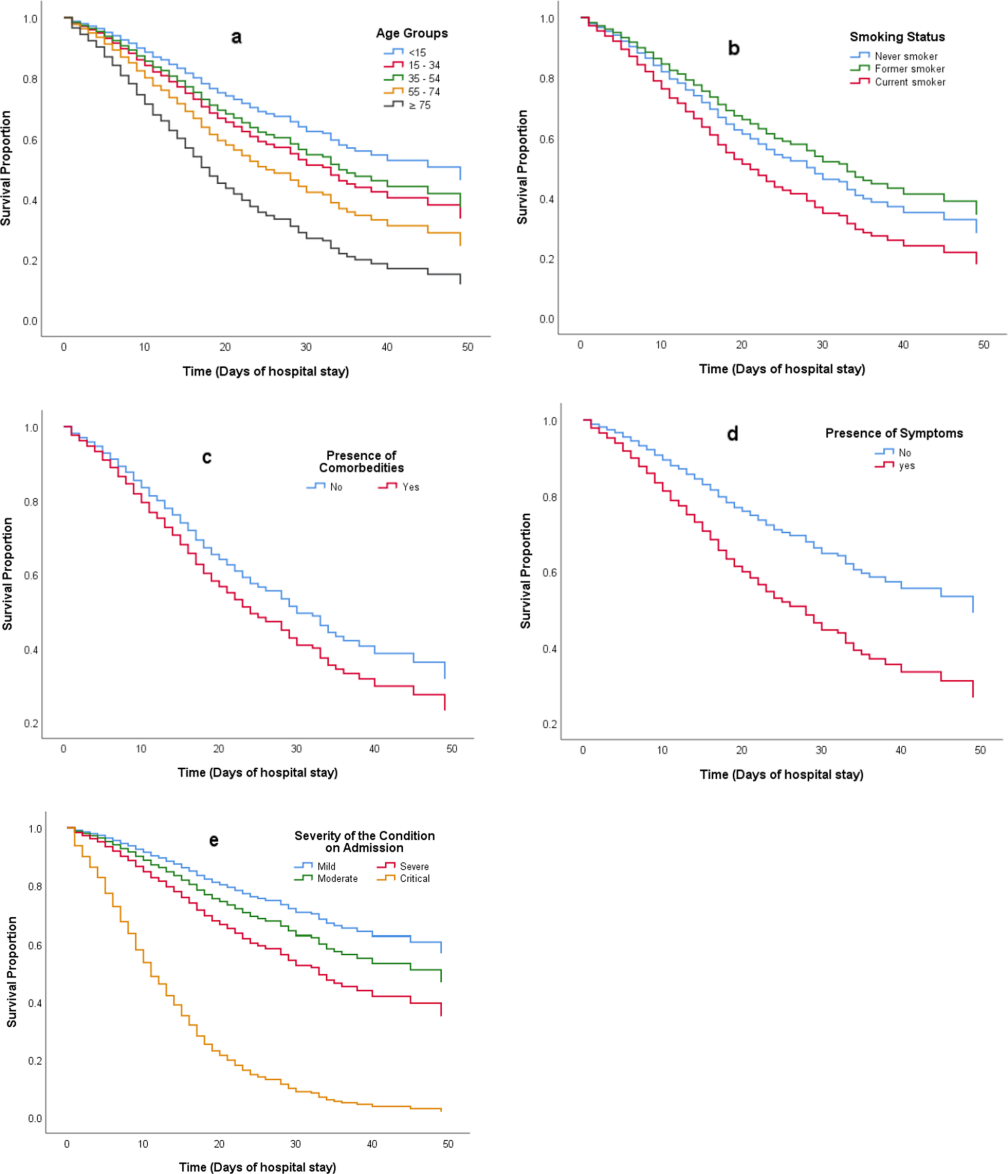
Kaplan–Meier curves for cumulative survival of COVID-19 patients stratified by age groups (**a**), smoking status (**b**), presence of comorbidities (**c**), presence of symptoms (**d**), and the severity of patients’ condition on admission (**e**)

Bivariate analysis of mortality predictors is presented in Table 1 and Additional file 1: Table S1 and S2. On multivariable analysis (Table 2), independent mortality predictors were age, current smoking, severity of the condition on admission, and the presence of comorbidities. Mortality risk in patients aged 55–74 years and ≥ 75 years was nearly double (HR 1.80, 95% CI 1.26–2.58) and triple (HR 2.74, 95% CI 1.90–4.00) that among patients aged < 15 years respectively. Current smoking increased mortality risk among smokers by 38% compared to never smokers (HR 1.38, 95% CI 1.07–1.77); and the presence of comorbidities increased it by 28% (HR 1.28, 95% CI 1.12–1.46). Also, the risk of mortality increased when patients admitted in severe (HR 1.93, 95% CI 1.30–2.87) or critical condition (HR 7.19, 95% CI 4.88–10.58) compared to asymptomatic/mild one. Kaplan–Meier survival curve showed decreased patients’ survival with the previously mentioned characteristics. Early separation from the reference categories was shown for categories representing patients who were aged ≥ 75 years, symptomatic, and admitted in critical condition that indicates rapid mortality onset (Fig. 3).

**Table 2:** Independent mortality predictors among total sample (n = 3663): demographics and clinical characteristics

	Mortality incidence	Adjusted HR^a^ (95% CI)	*P*-value
	% (deaths/total)		
*Age*			
< 15	19.4 (32/165)	Ref	
15–34	12.2 (61/498)	1.39 (0.90–2.14)	0.142
35–54	15.5 (141/912)	1.26 (0.85–1.85)	0.246
55–74	30.3 (490/1618)	1.80 (1.26–2.58)	**0.001**
≥ 75	52.8 (248/470)	2.74 (1.90–4.00)	** < 0.001**
*Gender*			
Female	25.8 (438/1698)	Ref	
Male	27.2 (534/1965)	0.97 (0.85–1.11)	0.660
*Tobacco use*			
Never	25.7 (877/3419)	Ref	
Former	37.8 (28/74)	0.83 (0.56–1.22)	0.333
Current	39.4 (67/170)	1.38 (1.07–1.77)	**0.014**
*Condition on admission*			
Asymptomatic/Mild	7.9 (29/368)	Ref	
Moderate	10.6 (97/911)	1.35 (0.89–2.06)	0.162
Severe	16.4 (238/1447)	1.93 (1.30–2.87)	**0.001**
Critical	64.9 (608/937)	7.19 (4.88–10.58)	** < 0.001**
*Presence of comorbidities*			
No	19.1 (384/2014)	Ref	
Yes	35.7 (588/1649)	1.28 (1.12–1.46)	** < 0.001**

*HR* Hazard ratio, *CI* Confidence interval

Bold indicates statistical significance

^a^Multivariable Cox proportional hazard regression was used. Variables included were age, gender, tobacco use, condition on admission, and presence of comorbidities

The effect of each comorbid condition on COVID-19 mortality corrected by age, gender, and smoking status was shown in Table 3 (Model 1). Additionally, accounting for the severity of the condition on admission and the simultaneous presence of comorbidities was done to determine the independent mortality predictors (Table 3 Model 2). Comorbidities that independently increased mortality risk were obesity (HR 1.39, 95% CI 1.08–1.79), malignancy (HR 1.84, 95% CI 1.33–2.53), and chronic haematological disorders (HR 1.68, 95% CI 1.08–2.61). An increased risk was also observed in patients presented with dyspnoea (HR 3.73, 95% CI 2.97–4.68) and respiratory distress/hypoxia (HR 3.65, 95% CI 2.90–4.60).

**Table 3:** Independent mortality predictors: specific comorbidities and clinical presentation (n = 3663)

	Mortality incidence	Adjusted HR (95% CI)^a^	*P*-value	Adjusted HR (95% CI)^a^	*P*-value
	% (deaths/total)	Model 1^b^		Model 2^c^	
*Specific comorbidities*					
Hypertension	34.5 (364/1055)	1.24 (1.09–1.42)	**0.001**	1.09 (0.91–1.30)	0.347
Diabetes	32.1 (321/999)	1.14 (1.0–1.31)	0.055	0.94 (0.78–1.12)	0.476
Heart disease (other than ischemic)	47.1 (101/213)	1.44 (1.17–1.78)	**0.001**	0.98 (0.78–1.24)	0.879
Obesity	41.8 (81/194)	1.60 (1.28–2.01)	** < 0.001**	1.39 (1.08–1.79)	**0.011**
Ischemic heart disease	38.5 (60/156)	1.37 (1.05–1.78)	**0.019**	1.21 (0.92–1.58)	0.169
Chronic kidney disease	46 (58/126)	1.49 (1.14–1.96)	**0.004**	1.03 (0.78–1.36)	0.829
Cerebrovascular disease	48.7 (57/117)	1.47 (1.13–1.93)	**0.005**	0.99 (0.74–1.31)	0.923
Chronic liver disease	54.7 (47/86)	1.51 (1.12–2.03)	**0.007**	0.84 (0.62–1.13)	0.25
Malignancy	56.6 (43/76)	2.37 (1.72–3.25)	** < 0.001**	1.84 (1.33–2.53)	** < 0.001**
Chronic obstructive pulmonary disease	44.6 (33/74)	1.91 (1.35–2.71)	** < 0.001**	1.24 (0.86–1.77)	0.251
Surgery	40.7 (24/59)	1.76 (1.17–2.66)	**0.007**	1.12 (0.73–1.70)	0.611
Other lung diseases	41.1 (23/56)	1.85 (1.22–2.80)	**0.004**	1.04 (0.68–1.59)	0.855
Chronic hematological disease	41.2 (21/51)	2.31 (1.50–3.57)	** < 0.001**	1.68 (1.08–2.61)	**0.022**
Immunological disorder	33.3 (14/42)	1.86 (1.09–3.15)	**0.022**	1.64 (0.86–3.13)	0.133
Transplant recipients	40.9 (9/22)	1.85 (0.96–3.57)	0.068	1.54 (0.63–3.78)	0.348
Pregnancy	50.0 (5/10)	1.36 (0.56–3.28)	0.499	0.50 (0.17–1.44)	0.201
*Specific symptoms/presentation*^d^					
Dyspnea	30.5 (399/1307)	1.32 (1.16–1.51)	** < 0.001**	3.73 (2.97–4.68)	** < 0.001**
Distress/Hypoxia	44.2 (559/1265)	2.14 (1.88–2.43)	** < 0.001**	3.65 (2.90–4.60)	** < 0.001**
Diarrhea	42.7 (177/415)	1.49 (1.26–1.75)	** < 0.001**	1.00 (0.84–1.19)	0.987

*HR* Hazard ratio, *CI* Confidence interval

Bold indicates statistical significance

^a^Multivariable Cox proportional hazard regression was used

^b^Model 1: Each comorbidity or symptom was separately corrected by age, gender, and smoking status

^c^Model 2: As Model-1 with further correction by the condition on admission, and the presence of other comorbidities or symptoms

^d^Variables included were those with *P*-value < 0.05 on bivariate analysis

#### Biomarkers

A detailed description of all tested biomarkers and their effect on mortality risk after accounting for age, gender, and smoking is presented in Additional file 1: Table S3. Mortality risk increased in patients with elevated C-reactive protein [8–10 mg/L (HR 1.32, 95% CI 1.01–1.72) and > 100 mg/L (HR 2.27, 95% CI 1.72–3.01)], serum ferritin [> normal–500 ng/mL (HR 1.75, 95% CI 1.28–2.39) and > 1000 ng/mL (HR 1.51, 95% CI 1.12–2.03)], Lactate Dehydrogenase [> 1000 unites/L (HR 2.05, 95% CI 1.28–3.31), INR [1.2– < 1.5 (HR 1.59, 95% CI 1.19–2.13) and 1.5– < 3 (HR 2.05, 95% CI 1.51–2.79)], and D-dimer [0.5– < 1 mcg/mL (HR 6.90, 95% CI 4.20–11.33) 1– < 4 mcg/L (HR 9.24, 95% CI 5.82–14.68) ≥ 4 mcg/L (HR 7.14, 95% CI 4.38–11.64). Other mortality predictors were anaemia (HR 1.26, 95% CI 1.06–1.49), leucocytosis (HR 1.98, 95% CI 1.70–2.30), thrombocytopenia (HR 1.25, 95% CI 1.06–1.49), and biomarkers indicative of impaired renal or hepatic function and disturbed electrolyte levels.

### Prognostic models

**Fig. 4:**
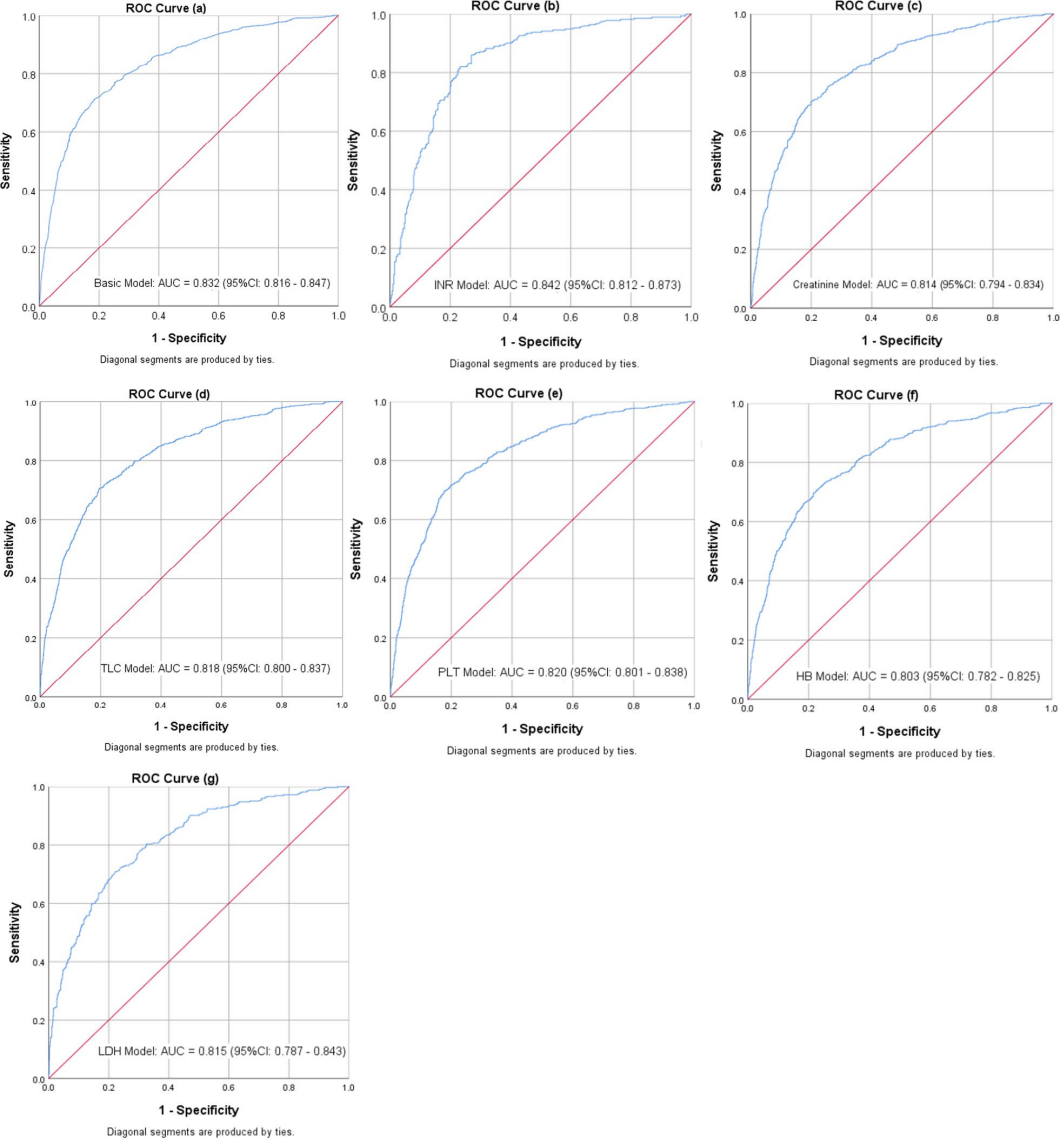
ROC Curves for Basic (**a**), INR (**b**), creatinine (**c**), TLC (**d**), PLT (**e**), HB (**f**), and LDH (**g**) Models. *INR* International normalized ratio, *TLC* Total leucocytic count, *PLT* Platelet count, *HB* Haemoglobin, *LDH* Lactate dehydrogenase

**Fig. 5:**
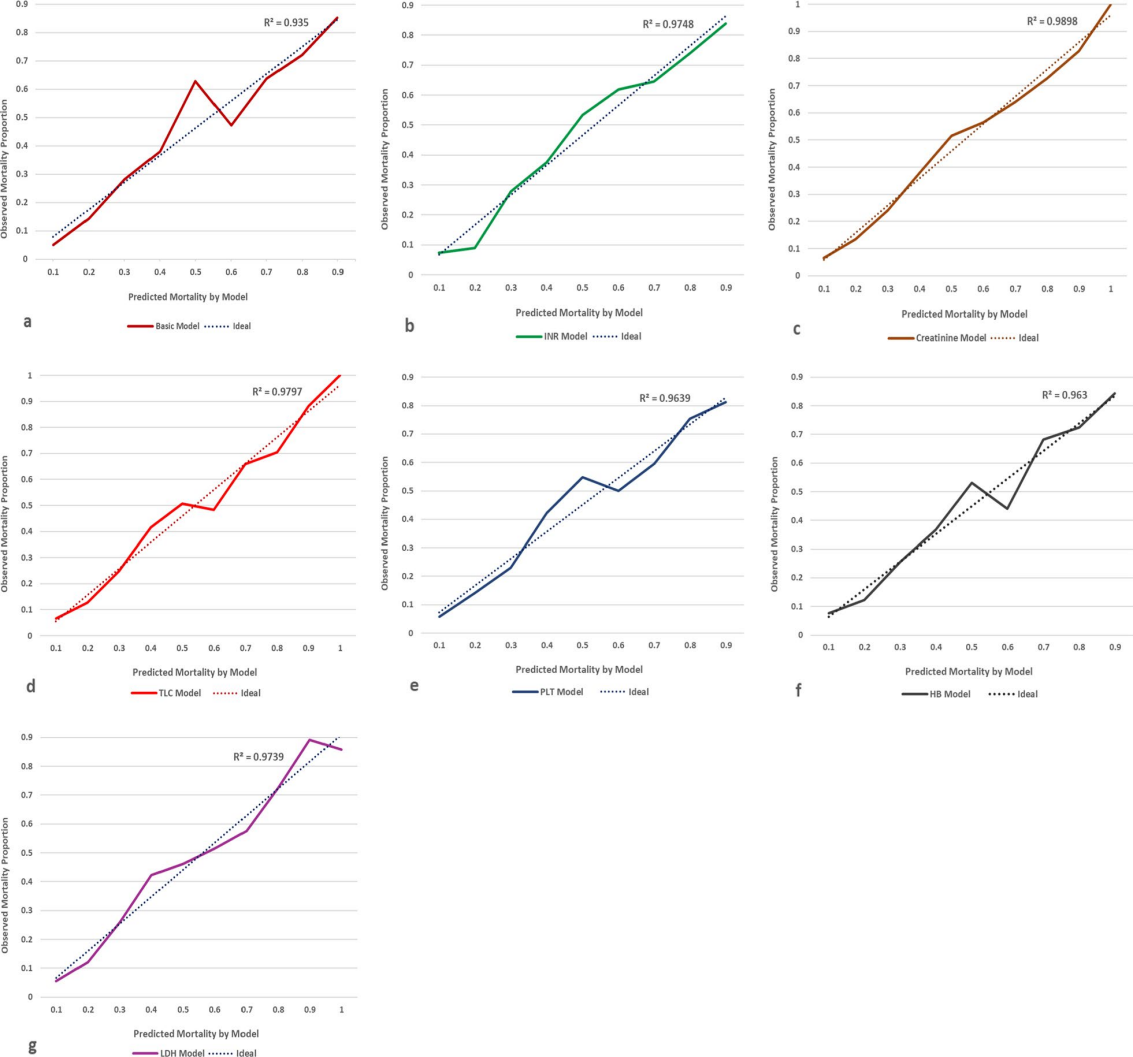
Calibration plot for Basic (**a**), INR (**b**), creatinine (**c**), TLC (**d**), PLT (**e**), HB (**f**), and LDH (**g**) Models. *INR* international normalized ratio, *TLC* Total leucocytic count, *PLT* Platelet count, *HB* Haemoglobin, *LDH* Lactate dehydrogenase

The basic model and additional 10 models corresponding to the added biomarkers were presented in Table 4 ordered by their balanced accuracy. Models with the highest balanced accuracy and AUC were the model containing International Normalized Ratio (INR) [77.8% and 0.842 (0.812–0.873) respectively] followed by the basic model [72.8% and 0.832 (0.816–0.847) respectively] (Fig. 4). Calibration plot showed better performance for INR model than the basic one (Fig. 5) and a good model fit for the data (HL test *P*-value 0.982). Also, models with creatinine, total leucocytic count, platelet count, haemoglobin level, and Lactate Dehydrogenase (LDH) had balance accuracy of ≥ 70% and AUC > 0.80; and their calibration plots were presented (Table 4 and Figs. 4, 5). Details of parameters for each model were presented in Additional file 1: Table S4. Using these parameters, calculation of a patient’s predicted mortality probability can be done [equations with examples are supplied in the supplementary materials (Additional file 1)]. For the basic and INR models, selected sensitivity values were presented with their associated specificity and predicted probability cut-off values. Reducing the cut-off values improved model sensitivity without much reduction in specificity (Table 5). The extra models containing individual comorbidities namely obesity, chronic haematological disease, and malignancy; and the model with the added smoking status showed almost the same performance as the basic model (Table 4).

**Table 4:** Models with the best performance among all tested models

Models	Number (death/total)	AUC (95%CI)	Sensitivity (%)	Specificity (%)	PPV (%)	NPV (%)	Accuracy (%)		HL *P*-value
							Overall	Balanced	
*Basic and biomarkers’ models*									
Basic Model	972/3663	0.832 (0.816–0.847)	55.0	90.6	67.8	84.8	81.1	72.8	0.010
INR	272/668	0.842 (0.812–0.873)	75.7	79.8	72.0	82.7	78.1	77.8	0.982
Creatinine	658/1980	0.814 (0.794–0.834)	56.2	87.8	69.7	80.1	77.3	72.0	0.583
TLC	745/2429	0.818 (0.800–0.837)	54.1	88.8	68.2	81.4	78.2	71.5	0.597
PLT	724/2340	0.820 (0.801–0.838)	53.2	88.6	67.7	80.9	77.6	70.9	0.549
HB	615/1813	0.803 (0.782–0.825)	53.7	87.9	69.5	78.7	76.3	70.8	0.093
LDH	326/1026	0.815 (0.787–0.843)	50.6	89.4	69.0	79.5	77.1	70.0	0.783
Troponin	112/285	0.747 (0.689–0.805)	54.5	84.4	69.3	74.1	72.6	69.5	0.671
CRP	538/1872	0.799 (0.777–0.821)	47.6	90.8	67.5	81.1	78.4	69.2	0.575
CK-Total	205/615	0.739 (0.697–0.781)	42.0	87.6	62.8	75.1	72.4	64.8	0.597
CK-MB	157/435	0.729 (0.680–0.777)	45.2	84.2	61.7	73.1	70.1	64.7	0.975
*Extra models*									
Smoking	972/3663	0.832 (0.817–0.847	55.0	90.6	67.9	84.8	81.2	72.8	0.029
Obesity	972/3663	0.830 (0.814–0.845)	55.3	90.5	67.8	84.9	81.2	72.9	0.005
Haematological disease	972/3663	0.829 (0.814–0.845)	55.3	90.4	67.5	84.8	81.1	72.9	0.0004
Malignancy	972/3663	0.831 (0.816–0.846)	56.3	90.3	67.7	85.1	81.3	73.3	0.004

Basic Model included: Age, Comorbidity presence, and Condition on admission. Each of the biomarker’s models and Smoking model included variables of basic model + the variable of the corresponding biomarker or smoking status. Obesity, Haematological, and Malignancy models included Age, Condition on admission, and the corresponding comorbidity variable

Binary logistic regression was used to calculate the model parameters then, death predicted probability was calculated for each model

*AUC* Area under the ROC curve, *PPV* Positive predictive value, *NPV* Negative predictive value, *HL* Hosmer–Lemeshow test, *INR* International normalized ratio, *TLC* Total leucocytic count, *PLT* Platelet count, *HB* Haemoglobin, *LDH* Lactate dehydrogenase, *CRP* C-reactive protein, *CK* Creatine kinase. CK-MB Creatine kinase-myoglobin binding

**Table 5:** Basic and INR models: Selected model sensitivity values and the corresponding specificity and cut-off values of predicted mortality probability

Sensitivity (%)	Basic model		INR model	
	Specificity (%)	Predicted probability (%)	Specificity (%)	Predicted probability (%)
85	62.5	16.0	73.0	29.3
80	70.7	18.5	77.5	35.9
75	75.8	20.7	80.1	52.0
70	82.3	24.1	83.6	62.4
65	86.3	28.2	85.4	66.9
60	89.3	37.0	85.9	69.1
55	90.6	50.0	88.1	70.3

*INR* International normalized ratio

## Discussion

### Main findings

This study presented one of the largest cohorts of hospitalized COVID-19 patients in our region. Mostly, patients were admitted to our hospitals as a referral from the Ministry of Health hotline referral system. Two thirds of patients admitted in severe or critical condition and almost half of them suffered pre-existing comorbidities. Mortality was 26.5% (95% CI 25.1–28.0%) among total sample and 64.5% (95% CI 62.1–66.9%) among ICU admitted patients. Mortality predictors were older age, current smoking, admission in severe and critical conditions, the presence of comorbidities, presenting with dyspnoea or respiratory distress/hypoxia, elevation of inflammatory and coagulation biomarkers, and disturbance of haematological, hepatic, and renal biomarkers. Rapid onset of death was specifically observed with elderly (≥ 75 years), symptomatic, and patients admitted in critical condition. Prognostic models depending mainly on clinical and radiological findings showed high accuracy in predicting mortality.

### In-hospital mortality

The in-hospital mortality of patients with COVID-19 varied widely by geographic region and by the level of patients’ care. The all-cause mortality among hospitalized patients was 37% (95% CI 25–51%) in China, 55% (95% CI 50–59%) in Asia, 26% (95% CI 26–27%) in Europe, 24% (95% CI 11–46%) in Americas, and the pooled rate was 32% (95% CI 23–43%). Among ICU admitted patients, all-cause mortality was 39% (95% CI 28–52%) in China, 48% (95% CI 13–85%) in Asia, 34% (95% CI 28–40%) in Europe, 15% (95% CI 10–23%) in Americas, 39% (95% CI 20–62%) in the Middle East, and the pooled rate was 35% (95% CI 28–43%) [14]. Although mortality among our hospitalized patients resembled that reported in Europe, Americas, and the pooled estimate; our ICU mortality was comparable with the regions reported high rates. It is to be noted that patients admitted to the ICU constituted a large proportion (41.1%) of our cohort. Many factors might have contributed to the relatively high ICU mortality. First, because early seeking for medical care is not a norm in our society and hospital isolation is not a preferred choice [23], and patients usually presented late after trying some of the over-the-counter treatments. Second, due to stretching and exhaustion of the healthcare capacity in the epidemic, a considerable proportion of our patients were admitted as a referral from other healthcare facilities; a condition that probably added more delay to their presentation. Third, with increased demand of ICU admission in the peak of epidemic that—on many occasions—exceeded our ICU bed capacity, the treating physicians were obliged to manage some severe patients (that were candidates for ICU in the usual conditions) in the intermediate care beds and conserve the precious ICU beds for the more critical cases.

### Mortality predictors

Literature provided strong evidence that older age patients were at higher risk of COVID-19 mortality [4, 13, 24]. Likewise, our results provided another supportive evidence for this association. The age-related poor outcome might be due to the chronic condition commonly associated with age, the low level of immunity, [25] the impaired response to viral infection due to the reduction in T-cell and B-cell clonal diversity [26], or the prolonged proinflammatory immune response [27]. Regarding male gender, despite reporting its association with higher mortality risk in many studies including meta-analyses [4, 13, 28] no gender difference was observed in some studies [13, 18, 29]. Similarly, our results did not show difference in mortality risk between gender.

Although tobacco/nicotine use was not associated with higher odds of SARS-CoV-2 positive test results in non-hospitalized individuals [30], current smoking increased the disease severity and doubled the risk of COVID-19 mortality compared to non-smokers among hospitalized patients [24, 28, 31, 32]. Confirming this finding, a 38% increase in mortality risk was found among current smokers compared with never smokers. A hypothesized mechanism for the adverse outcome is that smoking increases the pulmonary expression of the angiotensin-converting enzyme-2 that suggested an increased risk of SARS-CoV-2 cell binding and entry [33].

Admission in critical condition, and the presence of comorbidities are predictor of poor patients’ outcome including death [4]. The increased mortality risk associated with the presence of comorbidities was documented in studies with high to moderate certainty evidence [4, 13, 28, 34]. There was growing evidence for the increased risk of infection, disease severity, hospitalization, ICU admission, and mortality in obese patients that was independent of the presence of other comorbidities [24, 35, 36]. Our results confirmed the independence of obesity as a COVID-19 mortality predictor. However, the mechanism underlying the association of obesity with the severe COVID-19 outcome remains unclear [36]. Malignancy was associated with increased mortality risk [24] that was highest with haematological types. Our data also demonstrated higher mortality in patients with malignancy. This association is probably due to the immunosuppression required for treatment [37]. Limited evidence suggested that patients with haemoglobin disorders were at increased risk of severe disease [38]. In the UK, a large cohort study demonstrated an increased hospitalization and COVID-19 mortality risk in presence of sickle cell disease and trait [39], for whom infection can cause acute chest syndrome [40]. In line with this finding, our data showed increased mortality risk in patients with haematological disorders.

Other chronic conditions with evidence of increased COVID-19 mortality were hypertension (independence of association was unclear), diabetes, heart disease, CKD, cerebrovascular disease, COPD (controversial evidence), chronic liver disease (limited evidence), immunological disorders (limited evidence), and surgery (limited evidence) [4, 13, 28]. Our results showed the increased COVID-19 mortality risk in association with the abovementioned conditions in models adjusting each condition with age, gender, and smoking status. However, their independence was not shown when additionally adjusted by the simultaneous presence of other comorbidities and the severity of patients’ condition on admission.

Patients presented with dyspnoea, respiratory distress, and hypoxia had higher COVID-19 mortality risk based on high certainty evidence [4, 28] and our results confirmed this finding.

A moderate certainty level of evidence was provided in support of blood/serum biomarker as predictors of COVID-19 mortality [9, 28]. Our results confirmed the increased mortality risk with the elevation of the inflammatory (C-reactive protein, serum ferritin, and LDH) and coagulation biomarkers (INR and D-dimer), anaemia, thrombocytopenia, leucocytosis, impaired hepatic and renal functions, and disturbance of electrolytes levels. Particularly, D-dimer elevation may be used as an indicator of clinical severity and poor patients’ outcome [34].

### Prognostic models

Many models were developed to predict in-hospital COVID-19 mortality based mainly on laboratory data. Hui Liu et al. created a risk score based on age and complete blood count (platelet counts, white blood cell, neutrophil, and neutrophil–lymphocyte ratio) [16]. Another scoring system that was developed on data from 452 severe COVID-19 patients included D-dimer, lymphopenia, procalcitonin, age > 60 years, and coronary heart disease [17]. The mortality score COVEG was also created and defined by age ≥ 54, neutrophil–lymphocyte ratio ≥ 2.88, D-dimer ≥ 0.795, C-reactive protein ≥ 30.1, serum ferritin ≥ 406, and the presence of cardiac diseases [41]. Also, a more complex model was built incorporating twelve patient’s characteristics that could challenge its practical applicability [15].

This study presented a group of prediction models based on the clinical evaluation that can help predict patients’ mortality risk. Models were built to be simple in containing the least possible number of relevant variables. For objectivity, the subjective patients’ history data were avoided as much as possible. The basic model contains items that will essentially be assessed for each patient (age, severity of the condition on admission, and comorbidity presence). The assessment of the severity of the condition on admission based mainly on clinical and radiological findings and optionally, on abnormal biomarkers (D-dimer, serum ferritin, lymphopenia, and liver functions). The addition of another biomarker in the other models helped testing the added value in mortality prediction attributed to such biomarker. Keeping the numeric variables during modelling, rather than turning them into categorical, was chosen to retain more predictive information. Only the INR model exhibited a better prediction than the basic one (sensitivity 75.7% vs. 55.0% respectively; balanced accuracy 77.8% vs. 72.8% respectively at 50% cut-off value for prediction probability). For these two models, presenting multiple cut-off probability values with their associated sensitivity and specificity were done for adaptability in clinical situations.

Our finding has a practical implication as it emphasized the importance of the severity of patients’ condition on admission in determining their outcome and underscored the rapid onset of death in elderly, symptomatic, and critical patients. Early patients’ presentation is specifically recommended for high-risk groups namely the elderly, current smoker, symptomatic, and those with any comorbidities specially obesity, malignancy, and haematological disorders. Highlighting smoking and obesity as mortality risk factors is important for taking the preventive and early treatment measures particularly in population with huge burden of both conditions like the case in Egypt (smoking 23%, overweight/obesity 63%, and obesity 36%) [42]. Being both modifiable risk factors, investment in programmes aiming to reduce weight and stop smoking could add another value in the era of pandemic. The supplied generic prognostic equations can simply be programmed for clinical bedside usage that adding a value as a clinical decision support tool.

## Strength and limitations

To our knowledge, this study represents one of the largest single-centre studies in our locality. The sample provided a sufficient power for estimating most of the mortality predictors and allowing for testing of their independence, hence adding evidence regarding controversial issues. Additionally, the sample enabled building several prognostic mortality prediction models. Being very generic, applicability of the provided models can also be tested with a non-COVID-19 respiratory infection.

Our reported mortality rate should be interpreted within the context of the case severity mix of our sample that was deviated towards including many patients (65.1%) in severe/critical conditions; a sample characteristic that entails cautious generalization of this result to other healthcare settings.

Underestimation of the frequency of the subjective and mild symptoms cannot be excluded. This could be attributed to recall and/or recording bias particularly with severe and critical patients who, due to their condition, were either unable to report or they overlooked such symptoms in favour of the more severe ones.

Smoking status may have many sources of information bias in hospital-based studies, as detailed smoking history is rarely taken. Commonly reported sources were the misclassification of former smokers and the underreporting of current smoking [43]. Smokers who suffered symptoms that warranted hospitalization and have recently quitted just before admission may correctly be classified as former smoker or misclassified as non-smoker. Their correct classification may inflate the mortality risk among former smokers and their misclassification may inflate it among non-smokers. Thus, the study end up with an inconclusive result regarding the mortality risk among former smokers compared to never smokers and also their observed higher survival—though insignificant—than never smokers. On the other hand, if misclassification of former smokers was accompanied by underreporting of current smoking, our observed effect size of current smoking on mortality risk may be underestimated.

Being a hospital-based study, determination of mortality predictors may be a subject of collider bias with possible inflation of associations [44]. Hence, mortality prediction based on the determined variables is best applicable among hospitalized COVID-19 patients rather than patients in the general population. Pragmatically however, patients with the identified predictors may still be consider as a priority group for the preventive and rapid treatment measures.

Our suggested prediction models were created on retrospective hospital-based data, making them dependant on the level of data accuracy. Being not externally validated, models require further testing for external validity in various clinical settings with similar and/or different case severity mix. Additionally, COVID-19 is an evolving phenomenon with changing epidemiology, level of herd immunity, and trending down overall mortality. This condition will mandate the need for external validation of the proposed models in a data set taken from a current population to ensure their continued usability in today’s situation.

## Conclusion

The risk of in-hospital COVID-19 mortality increases with older age, current smokers, and patients with a pre-existing comorbidity. Admission in a severe or critical condition is strongly associated with a fatal outcome. Out of an exhaustive list of comorbidities that showed evidence of increasing risk of COVID-19 mortality in some literature, only obesity, malignancy, and haematological disorders independently increased this risk. Pragmatically, patients with the identified predictors are to be prioritized for preventive and rapid treatment measures. Early seeking of medical care is recommended particularly in high-risk patients. Targeting the issues of smoking and body weight for preventive and treatment programs may add a value in the era of COVID-19 pandemic. Mortality prediction models with high accuracy were built for clinical usage. With the models provided, clinicians can calculate mortality probability for their patients. Presenting multiple simple and very generic models can enable clinicians to choose the model containing the parameters available in their specific clinical setting, and also to test the applicability of such models in non-COVID-19 respiratory infection.

## Supplementary Information

**Additional file 1:** **Supplementary Table 1.** Comorbidities: proportions and mortality prediction. **Supplementary Table 2.** Presenting symptoms/conditions: proportions and mortality prediction. **Supplementary Table 3.** Biomarkers as mortality predictors. **Supplementary Table 4.** Details of model parameters: intercept and β coefficients for model components. **Predicted mortality probability:** equations and example.

## Data Availability

The data underlying this article will be shared on reasonable request to the corresponding author.

## Abbreviations

COVID-19: Corona virus disease 2019
SARS-CoV-2: Severe acute respiratory syndrome coronavirus 2
ASUHs: Ain Shams University Hospitals
HRCT: High resolution computed tomography
RT–PCR: Reverse transcription–polymerase chain reaction
ICU: Intensive care unit
CORAD: COVID-19 Reporting and Data System
COPD: Chronic obstructive pulmonary disease
CKD: Chronic kidney disease
IQR: Interquartile range
HR: Hazard ratio
CI: Confidence interval
ROC: Receiver operating characteristic
AUC: Area under the ROC curve
HR: Hosmer–Lemeshow test
INR: International normalized ratio
LDH: Lactate dehydrogenase
