# Supplementary material for: Developing a mortality risk prediction model using data of 3663 hospitalized COVID-19 patients: a retrospective cohort study in an Egyptian University Hospital
Source: BMC Pulm Med. 2023 Feb 7;23:57. doi: 10.1186/s12890-023-02345-3 (PMC9903412; doi:10.1186/s12890-023-02345-3)
Supplement: Supplementary file 1 — Additional file 1: Supplementary Table 1. Comorbidities: proportions and mortality prediction. Supplementary Table 2. Presenting symptoms/conditions: proportions and mortality prediction. Supplementary Table 3. Biomarkers as mortality predictors. Supplementary Table 4. Details of model parameters: intercept and β coefficients for model components. Predicted mortality probability: equations and example. [file 12890_2023_2345_MOESM1_ESM.pdf]

Supplementary Information

Article title: Developing a mortality risk prediction model using data of 3663 hospitalized COVID-19 patients: A retrospective cohort study in an Egyptian university hospital

Journal: *BMC Pulmonary Medicine*

Authors:

Sahar Kandil, [1] * M.D., Ayman I Tharwat,[2] M.D., Sherief M Mohsen,[3] M.D., Mai Eldeeb,[4] M.D., Waleed Abdallah,[2] M.D., Amr Hilal,[2] M.D., Hala Sweed,[5] M.D., Mohamed Mortada,[5] M.D., Elham Arif,[5] M.D., Tarek Ahmed,[4] M.D., Ahmed Elshafie,[4] M.D., Tarek Youssef,[3] M.D., Mohamed Zaki,[3] M.D., Yasmin El-Gendy,[6] M.D., Essam Ebied,[3] M.D., Safwat Hamad,[7] Ph.D., Ihab Habil,[1] M.D., Hany Dabbous,[8] M.D., Amr El-Said,[2] M.D., Yasser Mostafa,[9] M.D., Samia Girgis,[10] M.D., Ossama Mansour,[11] M.D., Ali El-Anwar,[3] M.D., Ashraf Omar,[3] M.D., Ayman Saleh,[12] M.D., Mahmoud El-Meteini,[3,13] M.D.

[1] Department of Community, Environmental, and Occupational Medicine, Faculty of Medicine, Ain Shams University, Cairo, Egypt

[2] Department of Anaesthesia, Intensive Care and Pain Management, Faculty of Medicine, Ain Shams University, Cairo, Egypt

[3] Department of General Surgery, Faculty of Medicine, Ain Shams University, Cairo, Egypt

[4] Department of Internal Medicine, Faculty of Medicine, Ain Shams University, Cairo, Egypt

[5] Department of Geriatric Medicine and Gerontology, Faculty of Medicine, Ain Shams University, Cairo, Egypt

[6] Department of Paediatrics, Faculty of Medicine, Ain Shams University, Cairo, Egypt

[7] Department of Scientific Computing, Faculty of Computer and Information Sciences, Ain Shams University, Cairo, Egypt

[8] Department of Hepatology and Infectious Diseases, Faculty of Medicine, Ain Shams University, Cairo, Egypt

[9] Department of Chest Diseases, Faculty of Medicine, Ain Shams University, Cairo, Egypt

[10] Department of Clinical Pathology, Faculty of Medicine, Ain Shams University, Cairo, Egypt

[11] Department of Otolaryngology and Head and Neck Surgery, Faculty of Medicine, Ain Shams University, Cairo, Egypt

[12] Department of Cardiology, Faculty of Medicine, Ain Shams University, Cairo, Egypt

[13] Department of Hepatobiliary Surgery and Liver Transplantation, Ain Shams Centre for Organ Transplantation (ASCOT), Faculty of Medicine, Ain Shams University, Cairo, Egypt

***Corresponding Author**

Sahar Kandil, M.D. Department of Community, Environmental, and Occupational Medicine, Faculty of Medicine, Ain Shams University, Cairo, Egypt

https://orcid.org/0000-0001-9433-659X

Address: Faculty of Medicine, Ain Shams University, Cairo, Egypt, 38 Ramses St., Abbassia Square, Post code: 11566. **E-mail:** saharkandil@med.asu.edu.eg

Tel/Fax: +202 24346888

Supplementary Table 1: Comorbidities: proportions and mortality prediction

**Comorbidities**	**Total**	**Death**	**Unadjusted Hazard ratio (95% CI)** [a]	***P*-value**
	***n*=3663**	***n*=972 (26.5)**		
	***n* (column%)**	***n* (column%)**		
Hypertension	1055 (28.8)	364 (37.4)	1.41 (1.23-1.60)	**<0.001**
Diabetes	999 (27.3)	321 (33.0)	1.26 (1.10-1.44)	**0.001**
Heart disease (other than ischemic)	213 (5.8)	101 (10.4)	1.77 (1.44-2.18)	**<0.001**
Obesity	194 (5.3)	81 (8.3)	1.77 (1.41-2.22)	**<0.001**
Ischemic heart disease	156 (4.3)	60 (6.2)	1.47 (1.14-1.91)	**0.004**
Chronic kidney disease	126 (3.4)	58 (6.0)	1.60 (1.23-2.09)	**0.001**
Neurological disorder	117 (3.2)	57 (5.9)	1.74 (1.33-2.27)	**<0.001**
Chronic liver disease	86 (2.3)	47 (4.8)	1.69 (1.25-2.27)	**0.001**
Malignancy	76 (2.1)	43 (4.4)	2.19 (1.61-2.98)	**<0.001**
Chronic obstructive pulmonary disease	74 (2.0)	33 (3.4)	2.08 (1.47-2.95)	**<0.001**
Surgery	59 (1.6)	24 (2.5)	1.73 (1.15-2.60)	**0.008**
Other lung disease	56 (1.5)	23 (2.4)	1.87 (1.24-2.84)	**0.003**
Hematological disorders	51 (1.4)	21 (2.2)	1.85 (1.20-2.86)	**0.005**
Immunological disorders	42 (1.1)	14 (1.4)	1.69 (0.99-2.87)	0.051
Transplant recipients	22 (0.6)	9 (0.9)	1.99 (1.03-3.83)	**0.041**
Pregnancy [b]	10 (0.6)	5 (1.1)	1.79 (0.74-4.32)	0.196

[a] Bivariable Cox regression analysis

[b] Calculations among female group (total *n*=1698, non-survivors *n*=438)

Supplementary Table 2: Presenting symptoms/conditions: proportions and mortality prediction

	Total	Death	Unadjusted Hazard ratio (95% CI) [a]	*P*-value
	n=3663	*n*=972 (26.5)		
	n (column%)	*n* (column%)		
**Symptoms**				
Fever	2062 (56.3)	552 (56.8)	0.89 (0.79-1.01)	0.079
Cough	1525 (41.6)	391 (40.2)	0.93 (0.82-1.06)	0.252
Dyspnea	1307 (35.7)	399 (41.0)	1.44 (1.27-1.64)	**<0.001**
Distress/hypoxia	1265 (34.5)	559 (57.5)	2.40 (2.12-2.73)	**<0.001**
Diarrhea	415 (11.3)	177 (18.2)	1.61 (1.37-1.90)	**<0.001**
Malaise	263 (7.2)	67 (6.9)	0.97 (0.75-1.24)	0.785
Sore throat	105 (2.9)	27 (2.8)	0.94 (0.64-1.38)	0.752
Anosmia	47 (1.3)	17 (1.7)	1.41 (0.87-2.28)	0.161
Vomiting	11 (0.3)	1 (0.1)	0.32 (0.05-2.30)	0.260
Ageusia	9 (0.2)	2 (0.2)	0.97 (0.24-3.90)	0.968
Abdominal pain	8 (0.2)	1 (0.1)	0.40 (0.06-2.86)	0.363
**Complications**				
Disturbed conscious level	111 (3.0)	76 (7.8)	2.75 (2.18-3.48)	**<0.001**
Convulsions	28 (0.8)	9 (0.9)	1.13 (0.59-2.18)	0.718
Shock/arrest	21 (0.6)	20 (2.1)	7.85 (5.03-12.23)	**<0.001**
Acute kidney injury	17 (0.5)	10 (1.0)	2.86 (1.53-5.33)	**0.001**
Hemorrhage (any source)	17 (0.5)	8 (0.8)	2.03 (1.01-4.07)	**0.047**

[a] Bivariable Cox regression analysis

Supplementary Table 3: Biomarkers as mortality predictors

Biomarkers	Total	Death	Adjusted hazard ratio (95% CI) [a]	p-value	Mortality proportion (row %)
	***n*=3663**	***n*=972 (26.5)**			
	***n* (column%)**	***n* (column%)**			
**Inflammatory**					
C-Reactive protein	***n*=1872**	***n*=538**			
≤8	371 (19.8)	69 (12.8)	Ref.		18.6
>8 - 100	1067 (57.0)	284 (52.8)	1.32 (1.01-1.72)	**0.043**	26.6
>100	434 (23.2)	185 (34.4)	2.27 (1.72-3.01)	**<0.001**	42.6
Ferritin	***n*=1403**	***n*=404**			
Normal [b]	366 (26.1)	64 (15.8)	Ref.		17.5
> Normal - 500	321 (22.9)	119 (29.5)	1.75 (1.28-2.39)	**<0.001**	37.1
>500 - 1000	244 (17.4)	68 (16.8)	1.31 (0.93-1.86)	0.127	27.9
>1000	472 (33.6)	153 (37.9)	1.51 (1.12-2.03)	**0.007**	32.4
LDH	***n*=1026**	***n*=326**			
≤210	488 (47.6)	146 (44.8)	Ref.		29.9
>210 - 245	75 (7.3)	10 (3.1)	0.43 (0.22-0.81)	**0.009**	13.3
>245 - 1000	432 (42.1)	150 (46.0)	0.94 (0.74-1.19)	0.613	34.7
>1000	31 (3.0)	20 (6.1)	2.05 (1.28-3.31)	**0.003**	64.5
**Coagulation**					
INR	***n*=668**	***n*=272**			
<1.2	431 (64.5)	134 (49.3)	Ref.		31.1
1.2 - <1.5	120 (18.0)	73 (26.8)	1.59 (1.19-2.13)	**0.002**	60.8
1.5 - <3	103 (15.4)	60 (22.1)	2.05 (1.51-2.79)	**<0.001**	58.3
≥ 3	14 (2.1)	5 (1.8)	0.82 (0.34-2.02)	0.670	35.7
D-Dimer	***n*=1262**	***n*=389**			
<0.5	407 (32.3)	20 (5.1)	Ref.		4.9
0.5 - <1	212 (16.8)	75 (19.3)	6.90 (4.20-11.33)	**<0.001**	35.4
1 - <4	416 (33.0)	204 (52.4)	9.24 (5.82-14.68)	**<0.001**	49.0
≥ 4	227 (18.0)	90 (23.1)	7.14 (4.38-11.64)	**<0.001**	39.6
**Hematological**					
Hemoglobin	***n*=1813**	***n*=615**			
Normal	706 (38.9)	202 (32.8)	Ref.		28.6
Anemia	1046 (57.7)	391 (63.6)	1.26 (1.06-1.49)	**0.009**	37.4
Severe anemia	61 (3.4)	22 (3.6)	1.15 (0.74-1.79)	0.533	36.1
Total leucocytic count	***n*=2429**	***n*=745**			
Normal	1303 (53.6)	298 (40.0)	Ref.		22.9
Low	222 (9.1)	49 (6.6)	1.05 (0.77-1.42)	0.743	22.1
High	904 (37.2)	398 (53.4)	1.98 (1.70-2.30)	**<0.001**	44.0
Platelet	***n*=2340**	***n*=724**			
Normal	1812 (77.4)	521 (72.0)	Ref.		28.8
Low	440 (18.8)	181 (25.0)	1.25 (1.06-1.49)	**0.009**	41.1
High	88 (3.8)	22 (3.0)	1.02 (0.66-1.56)	0.943	25.0
**Renal and electrolytes**					
Creatinine	***n*=1980**	***n*=658**			
≤1.4	1137 (57.4)	296 (45.0)	Ref.		26.0
>1.4 - 6	726 (36.7)	314 (47.7)	1.76 (1.49-2.06)	**<0.001**	43.3
>6	117 (5.9)	48 (7.3)	1.60 (1.17-2.17)	**0.003**	41.0
Sodium	***n*=792**	***n*=304**			
135 - 145	469 (59.2)	176 (57.9)	Ref.		37.5
<135 - 120	267 (33.7)	87 (28.6)	0.87 (0.67-1.13)	0.288	32.6
<120	11 (1.4)	7 (2.3)	1.80 (0.84-3.90)	0.133	63.6
>145	45 (5.7)	34 (11.2)	3.03 (2.08-4.43)	**<0.001**	75.6

Potassium	***n*=748**	***n*=292**			
3.5 - 5.3	557 (74.5)	198 (67.8)	Ref.		35.5
<3.5 - 2.5	132 (17.6)	62 (21.2)	1.34 (1.100-1.79)	**0.048**	47.0
<2.5	8 (1.1)	3 (1.0)	1.02 (0.33-3.20)	0.972	37.5
>5.3 - 6	38 (5.1)	21 (7.2)	1.63 (1.03-2.57)	**0.036**	55.3
>6	13 (1.7)	8 (2.7)	3.58 (1.75-7.32)	**<0.001**	61.5
Calcium	***n*=575**	***n*=185**			
8.5 - 10.5	247 (43.0)	61 (33.0)	Ref.		24.7
<8.5	298 (51.8)	118 (63.8)	1.49 (1.09-2.04)	**0.014**	39.6
>10.5	30 (5.2)	6 (3.2)	1.22 (0.52-2.84)	0.645	20.0
Phosphorus	***n*=461**	***n*=167**			
3.4 - 4.5	141 (30.6)	42 (25.1)	Ref.		29.8
<3.4	195 (42.3)	63 (37.7)	0.79 (0.53-1.18)	0.240	32.3
>4.5	125 (27.1)	62 (37.1)	1.64 (1.09-2.47)	**0.017**	49.6
Magnesium	***n*=536**	***n*=211**			
1.8 - 2.2	268 (50.0)	94 (44.5)	Ref.		35.1
<1.8 - 1	70 (13.1)	28 (13.3)	0.88 (0.58-1.35)	0.567	40.0
>2.2	198 (36.9)	89 (42.2)	1.30 (0.97-1.74)	0.077	44.9
**Hepatic**					
Albumin	***n*=1783**	***n*=614**			
3.4 - 5.4	1001 (56.1)	269 (43.8)	Ref.		26.9
<3.4 - 2.4	633 (35.5)	258 (42.0)	1.38 (1.16-1.64)	**<0.001**	40.8
<2.4	149 (8.4)	87 (14.2)	2.32 (1.82-2.96)	**<0.001**	58.4
ALT	***n*=2116**	***n*=683**			
≤55	1756 (83.0)	537 (78.6)	Ref.		30.6
>55 - 165	302 (14.3)	118 (17.3)	1.23 (1.01-1.50)	**0.044**	39.1
>165 - 1000	49 (2.3)	23 (3.4)	2.12 (1.40-3.23)	**<0.001**	46.9
>1000	9 (0.4)	5 (0.7)	2.91 (1.20-7.04)	**0.018**	55.6
AST	***n*=2077**	***n*=670**			
≤48	1605 (77.3)	463 (69.1)	Ref.		28.8
>48 - 145	394 (19.0)	163 (24.3)	1.9 (1.25-1.79)	**<0.001**	41.4
>145 - 1000	68 (3.3)	36 (5.4)	1.68 (1.19-2.37	**0.003**	52.9
>1000	10 (0.5)	8 (1.2)	4.15 (2.03-8.47)	**<0.001**	80.0
**Cardiac**					
CK Total	**n=615**	**n=205**			
≤150	313 (50.9)	98 (47.8)	Ref.		31.3
>150 - 300	140 (22.8)	50 (24.4)	1.20 (0.85-1.69)	0.298	35.7
>300 - 1000	125 (20.3)	43 (21.0)	1.04 (0.72-1.50)	0.830	34.4
>1000	37 (6.0)	14 (6.8)	1.30 (0.74-2.30)	0.361	37.8
Troponin	**n=285**	**n=112**			
Normal	115 (40.4)	48 (42.9)	Ref.		41.7
>Normal - 2 Folds	50 (17.5)	15 (13.4)	0.93 (0.51-1.70)	0.820	30.0
> 2 Folds	120 (42.1)	49 (43.8)	1.15 (0.76-1.73)	0.521	40.8

[a] Cox regression analysis, accounting for age, gender, tobacco use, Each lab at a time
[b] Normal range: females 10 to 200 ng/mL; males 30 to 300 ng/mL
INR: International Normalized Ratio. LDH: Lactate Dehydrogenase.
CRP: C-Reactive Protein. AST: Aspartate aminotransferase. ALT: Alanine transaminase. CK: Creatine Kinase.
CK-MB: Creatine Kinase-Myoglobin Binding

Supplementary Table 4: Details of model parameters: intercept and β coefficients for model components

Models	Intercept	Age in years	Condition on admission				Presence of comorbidities		Added labs.
			Level (Coding value)				Level (Coding value)		Value
			Mild (0)	Moderate (1)	Severe (2)	Critical (3)	No (0)	Yes (1)	
**Basic Model**	-3.936	0.030	ref	-0.094	0.326	2.558	ref	0.431	
**Basic Model + INR**	-2.627	0.025	ref	-1.174	0.191	2.210	ref	-0.034	-0.106
**Basic Model + Creatinine**	-3.951	0.032	ref	-0.170	0.613	2.445	ref	0.192	0.074
**Basic Model + TLC**	-4.220	0.031	ref	-0.010	0.846	2.628	ref	0.183	0.250
**Basic Model + PLT**	-3.409	0.031	ref	0.123	0.863	2.687	ref	0.127	-0.002
**Basic Model + HB**	-3.450	0.031	ref	-0.014	0.775	2.524	ref	0.055	-0.018
**Basic Model + LDH**	-4.244	0.036	ref	-0.373	0.688	2.436	ref	-0.056	0.001
**Basic Model + Troponin**	-22.088	0.024	ref	19.856	20.244	21.582	ref	-0.672	0.001
**Basic Model + CRP**	-4.388	0.034	ref	0.379	1.011	2.651	ref	0.083	0.003
**Basic Model + CK-Total**	-2.897	0.022	ref	0.212	0.758	2.152	ref	-0.072	0.000004
**Basic Model + CK-MB**	-22.166	0.018	ref	20.020	20.505	21.565	ref	-0.328	0.005

Basic Model included: Age, Comorbidity presence, and Condition on admission
Binary logistic regression was used to calculate the model parameters.
INR: International Normalized Ratio. TLC: Total Leucocytic Count. PLT: Platelet count. HB: Haemoglobin. LDH: Lactate Dehydrogenase. CRP: C-Reactive Protein. CK: Creatine Kinase. CK-MB: Creatine Kinase-Myoglobin Binding.

Using the model parameters, calculation of a patient's predicted mortality probability can be done according to the following 2 simple equations (for the basic model as an example):

Predicted logit (Basic Model) = -3.936 + (0.030*Patient's Age) + (-0.094*Condition on Admmision_1) + (0.326*Condition on Admmision_2) + (2.558*Condition on Admmision_3) + (0.431*Comorbidity Presence).

Predicted mortality probability (Basic Model) = (2.718281828**predicted_logit_Basic_Model)/(1+2.718281828**predicted_logit_Basic_Model).

Example for a patient: age = 88, admitted in critical condition, and has comorbidities:

Predicted logit (Basic Model) = (-3.936) + (0.03*88) + (2.558*1) + (0.431*1) = 1.693

Predicted probability = 0.844618
